# IGFBPs were associated with stemness, inflammation, extracellular matrix remodeling and poor prognosis of low-grade glioma

**DOI:** 10.3389/fendo.2022.943300

**Published:** 2022-08-03

**Authors:** Zhihui Liu, Hang Ji, Wenchao Fu, Shuai Ma, Hongtao Zhao, Fang Wang, Jiawei Dong, Xiuwei Yan, Jiheng Zhang, Nan Wang, Jiasheng Wu, Shaoshan Hu

**Affiliations:** ^1^ Department of Neurosurgery, Cancer Center, Zhejiang Provincial People’s Hospital Affiliated to Hangzhou Medical College, Hangzhou, China; ^2^ Department of Neurosurgery, The Second Affiliated Hospital of Harbin Medical University, Harbin, China; ^3^ The Key Laboratory of Myocardial Ischemia, Ministry of Education, Harbin, China; ^4^ Department of Neurosurgery, West China Hospital, Sichuan University, Sichuan, China

**Keywords:** extracellular matrix, metabolism, stemness, inflammation, insulin-like growth factor binding protein (IGFBP)

## Abstract

**Background:**

The IGFBP family of insulin-like growth factor binding proteins has important biological functions in the organism. However, the role of the IGFBP family in low-grade glioma (LGG) has not been fully explored.

**Methods:**

We validated the clinical value of the IGFBP family using RNA-seq and clinical data of LGG in the TCGA and constructed an IGFBPScore using LASSO-regression analysis for prognosis prediction, subtype determination, and treatment sensitivity determination. Subsequently, we explored the role of the IGFBP family in the development of LGG using PanCanAtlas data.

**Results:**

Our results suggest that most IGFBP family members were aberrantly expressed and were strongly associated with poor prognosis in LGG. By constructing an IGFBPScore representing the IGFBP family, we found that tumor samples with a high IGFBPScore had a glioblastoma-like mutation pattern characterized by IDH1wt, EGFRmut, PTENmut, and NF1mut with hypo-methylation and glioma stem cell (GSC) diversity. In contrast, the low IGFBPScore group was characterized by IDH1mut accompanied by TP53mut, CICmut, and ATRXmut, and had hyper-methylation status as well as the GSC restriction. Additionally, the high-IGFBPScore group had a high inflammation phenotype with increased immune antigenicity and increased infiltration of immune molecules and cells, as well as a high extracellular matrix phenotype and enhanced multiple metabolic pathways compared with the immune-quiet phenotype of the low-IGFBPScore group, which was strongly associated with poor prognosis.

**Conclusion:**

Our study provides a summary analysis and a theoretical basis for the biological role and clinical value of the IGFBP family in LGG, providing an important therapeutic target for LGG.

## Introduction

Low-grade gliomas (LGGs) account for approximately 43.2% of CNS gliomas and include astrocytomas and oligodendroglial cell tumors. Current treatment for gliomas includes surgery, radiotherapy, and chemotherapy, but due to the high aggressiveness of LGG, the median survival time is only 78.1 months even with standard treatment (1). Immunotherapy presents new opportunities for tumor treatment because of its high selectivity and low adverse effects (2). However, the immunosuppressed and cold tumor state of the nervous system has led to the limitation of immunostimulation therapy in gliomas (3). Compared to other tumors, gliomas have special resident cells such as astrocytes, microglia, and neurons, as well as a special extracellular matrix (ECM) (4), and this special tumor microenvironment (TME) provides new perspectives for treating gliomas while affecting the effectiveness of immunotherapy.

The ECM is a protein network surrounding normal and cancer cells in the TME. Unlike other tissues rich in collagen and laminin, the composition of the brain ECM is unique, consisting mainly of glycoproteins, proteoglycans, and glycosaminoglycans (4). The binding of cells to the ECM is essential for many developmental processes and the maintenance of tissue homeostasis, and ECM remodeling is also necessary for cancer cells to invade the stromal tissue and become malignant (5). The dense ECM in gliomas leads to hypoxia and increased tumor aggressiveness, and as the cancer progresses, interactions between cancer cells and TME often lead to ECM stiffness, resulting in abnormal mechanotransduction, cancer cell proliferation, migration, angiogenesis, and genetic instability, which leads to resistance to radiotherapy and immunotherapy (6, 7). However, the regulation of ECM in LGG has not been well studied.

The IGFBP superprotein family is a group of ECM-regulated proteins, including IGFBP1–IGFBP7, which bind to circulating IGF receptors to reduce cell migration and cell adhesion to matrix proteins. The role of IGFBPs in tumors has recently received more attention. IGFBP is involved in transcriptional regulation, apoptosis promotion and DNA damage repair (DDR) by interacting with IGF receptors, glycosaminoglycans, integrins and other ligands, further affecting tumorigenesis, progression and drug resistance (8). IGFBP3 and IGFBP5 were overexpressed in gliomas and were associated with higher tumor grade and lower survival (9, 10). Upregulation of IGFBP2 expression was associated with angiogenic mimetic (VM) formation, leading to resistance to anti-VEGF therapy in glioma and had a significant immunosuppressive activity in GBM, which was negatively associated with patient survival (11). However, the role and possible mechanisms of the functionally important IGFBP protein family in LGG have not been fully explored.

Therefore, this paper aims to provide a comprehensive exploration of the function and mechanism of the IGFBP protein family in LGG. We first analyzed the abnormal expression of the IGFBP protein family in LGG and then determined the clinical value of IGFBP family proteins by their relationship with patient prognosis, clinical traits, molecular subtypes, and treatment response. Subsequently, we constructed a risk score system for the IGFBP family to predict prognosis as well as to determine the effect of treatment. Finally, we explored the mechanisms by which the IGFBP family affects the prognosis of LGG, including the relationship with the genome, epigenome, and glioma stem cells (GSCs), and the impact on the immune microenvironment, including the relationship with immune antigenicity, immune responsiveness, and immune evasion. Our study outlines the important role and mechanisms of the IGFBP family in LGG, providing new targets for personalized treatment of glioma.

## Material and methods

### Preparation of data

We obtained the RNA-seq and clinical data of LGG from the TCGA (The Cancer Genome Atlas, https://portal.gdc.cancer.gov/) and two cohorts (mRNAseq_325 and mRNAseq_693) of the CGGA (Chinese Glioma Genome Atlas, http://www.cgga.org.cn/). The RNA-seq data of the normal cerebral cortex (Brain–Cortex and Brain–Frontal Cortex (BA9)) were obtained from the GTEx database (https://www.gtexportal.org). The remaining clinical profiles, molecular subtypes, and genomic signatures of LGG were obtained from PanCanAtlas Publications (https://gdc.cancer.gov/about-data/publications/pancanatlas) and the MEXPRESS database (https://mexpress.be/index.html).

### Expression differences and prognostic analysis

We used 207 normal cerebral cortex tissues and 529 TCGA-LGG samples to perform the expression difference analysis using the R-loading “limma” package, and the difference heatmap was created using the “pheatmap” package. For survival analysis, survival time and survival status were recorded in the clinical data of 506 TCGA-LGG samples. The best cut-off was calculated using the “surv_cutpoint” function for grouping and using the R-loaded “survival” and “survminer” packages for survival analysis. The “survival” package was also used for univariate and multivariate COX analysis and visualization using the “forestplot” package.

### Construction and validation of the IGFBPScore

We created a risk score on account of the IGFBP family using “LASSO” regression for 1,000 iterations and calculated the risk score. IGFBPScore was calculated using the following equation: $IGFBPScore{=}_{i=1}^{n}Coef(IGFBPs_{i})*\exp(IGFBPs_{i})$ , where Coef refers to the regression coefficient and exp indicates gene expression. In the process of constructing the IGFBPScore, 70% of the samples were picked at random as train samples and the rest as test samples, and the risk curve of the IGFBPScore was plotted by the “pheatmap” package. Random forest validation using the “randomForest” package. To analyze the relationship between the IGFBPScore and the clinical traits, we calculated and plotted the clinical correlation heatmap using the “ComplexHeatmap” package, and a nomogram integrating the IGFBPScore and other clinical traits, as well as calibration plots, were plotted by the “rms” package.

### Single-sample gene-set enrichment analysis

The ssGSEA analysis was performed using “GSVA,” and 29 immune signatures were used to detect the immune functions and pathways (12). First, a matrix of all the gene expression values for LGG was prepared. Then, the gene sets corresponding to each biological process are obtained from the authoritative literature and prepared as gmt files, with the following main format: each row represents a biological process or pathway, the first column is the pathway ID, the second column is the description of the pathway, and the third to the last column is the gene of the pathway or biological process. Finally, the gene sets are quantified by the “GEVA” package using ssGSEA, which allows defining an enrichment score representing the absolute enrichment of gene sets in each sample within a given data set. The ssGESA score is normalized to a percentage distribution, where 0 is the minimum value of abundance and 1 is the maximum value. For comparison, we used the abundance values of biological processes from different samples. To discover the underlying mechanisms in different subgroups, typical biological processes were quantified by ssGSEA. We also introduced metabolic pathway gene sets, DDR gene sets, extracellular matrix structural component sets, immunogenic death, and EMT gene sets into our analysis, as shown in **Supplementary Table S1**.

### Acquisition of epigenetic data and stemness indices

The pan-glioma methylation subtype (13) was obtained from the reference. The “RColorBrewer” package was used to statistically analyze and visualize the proportion of methylation in high- and low-risk groups. Glioma stem cell markers were obtained from published single cell-seq files (14). The two stemness indices were obtained from the stemness of the tumor samples, in which mRNAsi reflects the gene expression characteristics of stem cells and mDNAsi reflects the epigenetic characteristics of stem cells. EREG-mRNAsi and EREG-mDNAsi were obtained by reconstructing the gene regulatory network from methylation and transcriptome data using the “ELMER” package and using the identified features as input to the “OCLR” (15). Stemness indices and stem cell markers were statistically analyzed using the “limma” package and visualized in box plots using “reshape2,” “ggplot2,” and “ggpubr.”

### Analysis of tumor microenvironment

Information on pan-cancer immunophenotype, leukocyte fraction (LF), immune molecules including stimulatory factors, inhibitory factors, HLA, immune checkpoint and antigenic peptide load were obtained from the literature (16). We used the “RColorBrewer” package to statistically analyze and visualize the immunophenotypes of the high- and low-risk groups, and the “limma” package to statistically analyze the differences between the high- and low-risk groups for the remaining immune parameters and to visualize them using “reshape2,” “ggplot2,” and “ggpubr.” The “CIBERSORT” package in R provides a deconvolution algorithm to quantify the 22 immuno-cells in the TME of each sample from the expression profile, and the “xcell” was used to calculate the stromal cells. The immune damage and escape, the response to immunotherapy, and the presence of CAF and MDSC were analyzed using the TIDE database: Tumor Immune Dysfunction and Exclusion (http://tide.dfci.harvard.edu/).

### Statistics

The above analyses were carried out by R version 4.0.2, Wilcoxon was used for the comparison of differences between the two cohorts, and Kruskal–Wallis test was used for comparison of differences among multiple groups. Survival curves were plotted using the Kaplan–Meier (K–M) method and Log-rank test was used to detect differences in survival between subgroups. Among the results calculated using all statistical methods above, p<0.05 was assessed as significant.

## Results

### Differential expression and clinical value of IGFBP family

**Figure 1:**
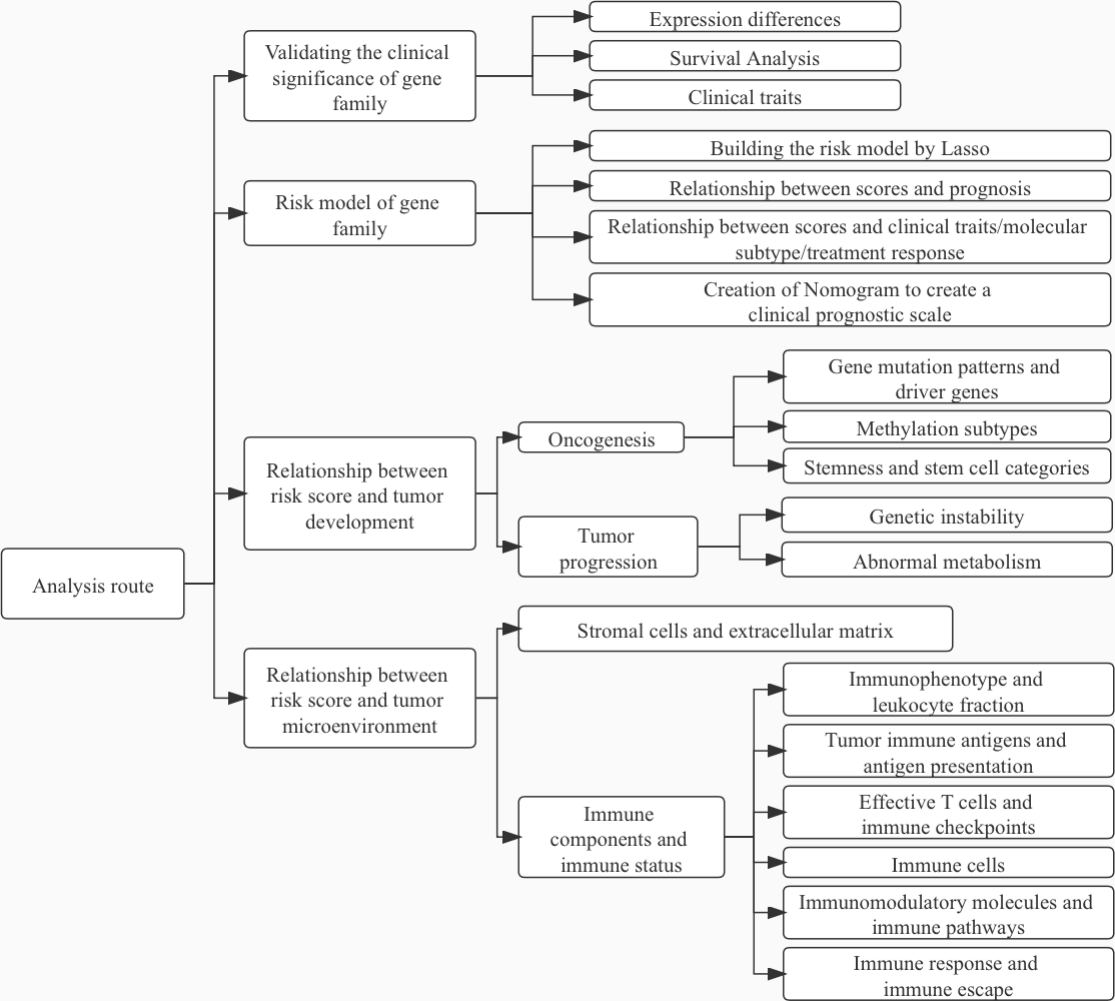
Article Analysis Flow Chart.

**Figure 2:**
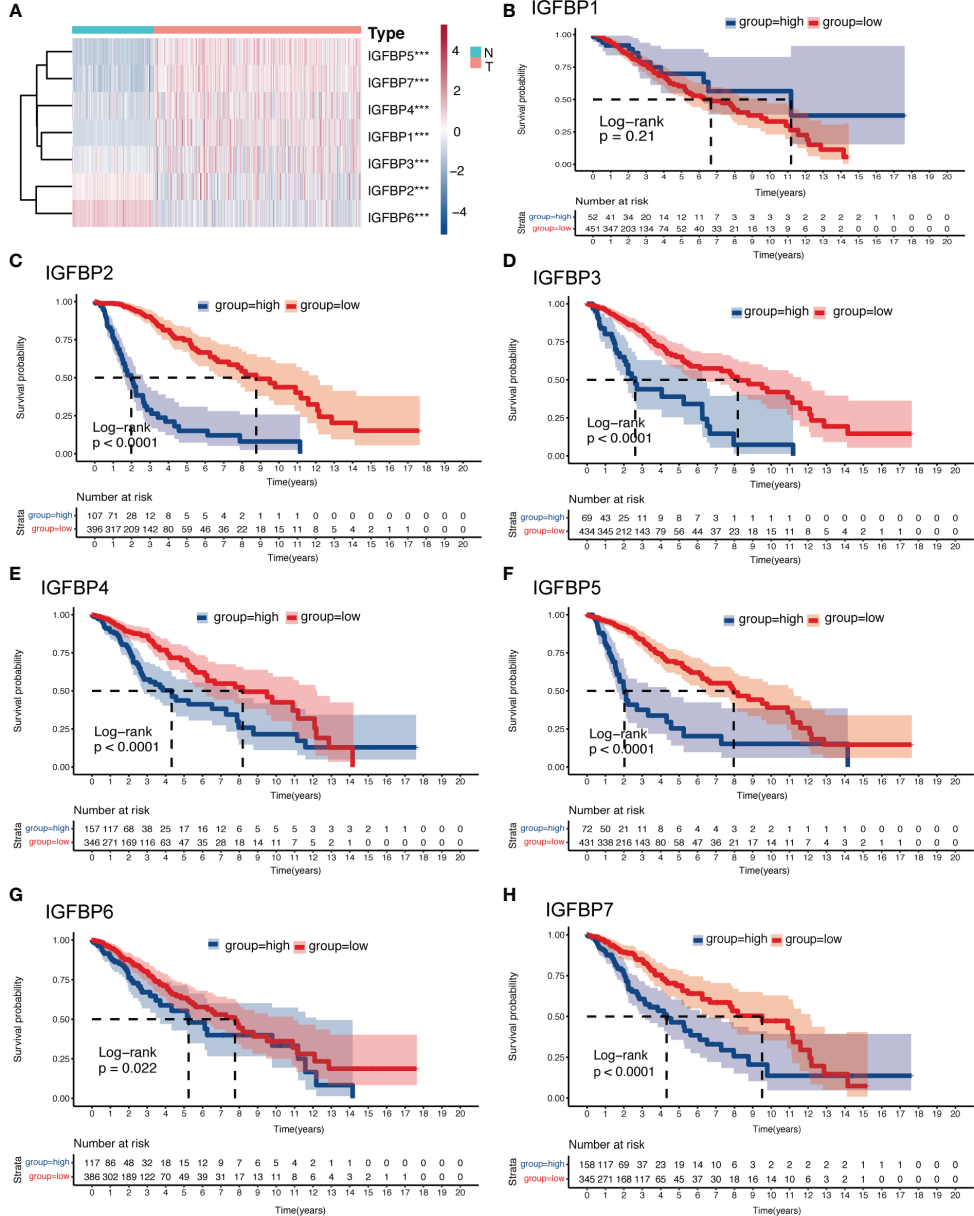
Expression and survival analysis of the IGFBP family of gliomas. **(A)** Heatmap of differential expression analysis of IGFBP family between normal cortex and the TCGA glioma cohort. **(B–H)** Kaplan–Meier survival analysis was performed using the optimal cutoff value to distinguish the IGFBP genes between high and low subgroups in the TCGA glioma cohort. For all experiments, ***p<0.001.

**Figure 3:**
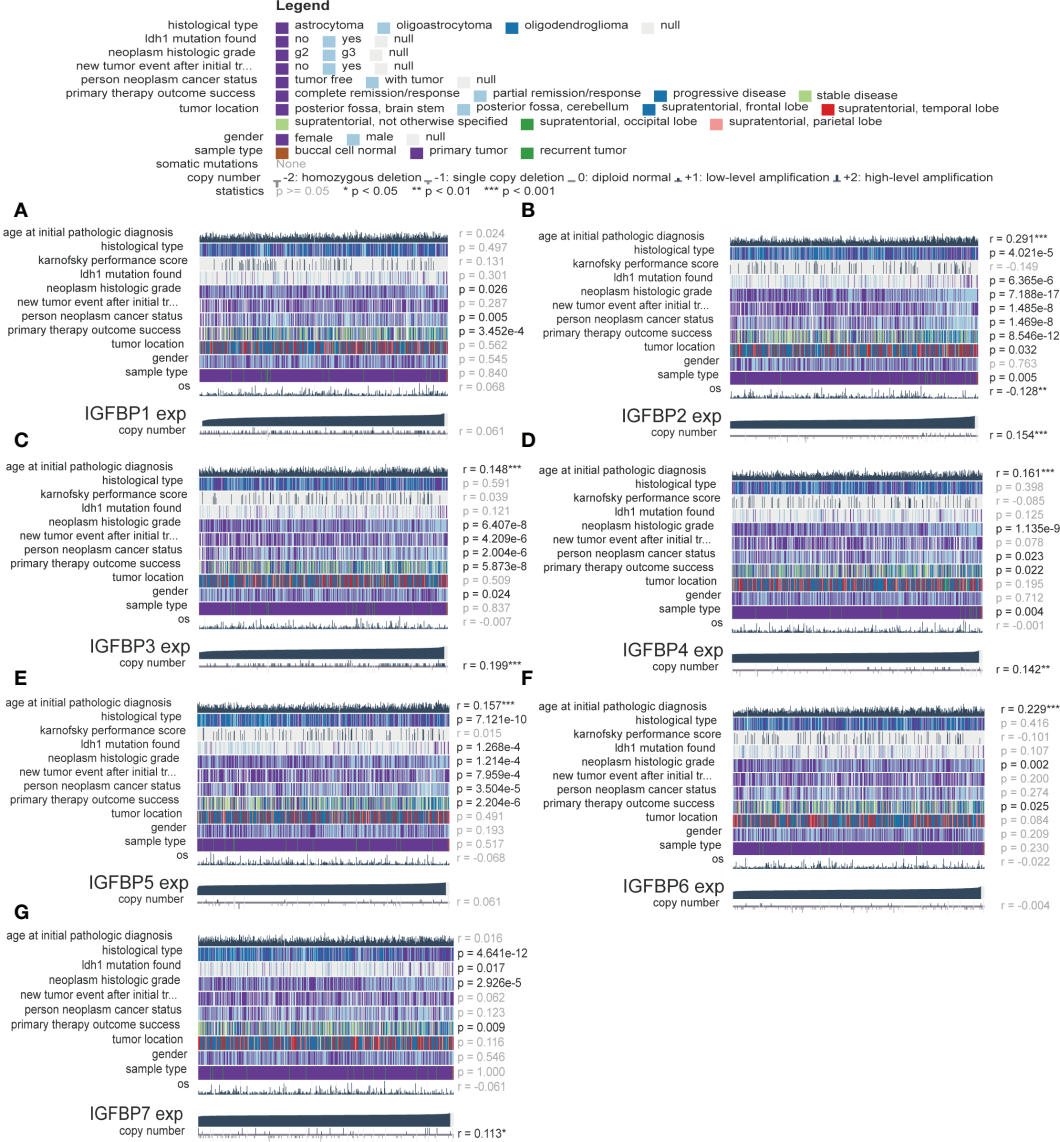
Validation of the clinical value of the IGFBP family. **(A–G)** Correlation heatmap of IGFBP family expression versus clinical traits in the glioma cohort. Each column in the graph represents a sample, while each row represents a variable. OS, overall survival. For all experiments, mean rank, *p<0.05, **p<0.01, ***p<0.001.

The analysis flow of this article is shown in **Figure 1**. To investigate whether IGFBP family proteins are dysregulated in LGG, we performed differential analysis of gene expression between the normal cortex and LGG. In **Figure 2A**, we found that the expression of all IGFBP family proteins differed significantly in LGG compared to normal brain tissue, with IGFBP1, IGFBP3, IGFBP4, IGFBP5, and IGFBP7 being significantly highly expressed in LGG, whereas IGFBP2 and IGFBP6 were significantly less expressed in LGG. But further analysis showed that the expression of all IGFBP proteins in the IDHwt subgroup was higher than that in the IDHmut subgroup (**Supplementary Figure S1A**). Subsequently, to investigate the effect of IGFBP proteins on the prognosis of LGG, we performed a survival analysis of IGFBP family genes in the TCGA cohort, as shown in **Figures 2B**–**H**. It was found that all classification of IGFBP family genes into high-exp and low-exp subgroups using optimal cutoff values, and it was found that all IGFBP expression except IGFBP1 was notably correlated with survival, and all showed shorter survival times in high-exp patients. Similar results were obtained using CGGA database validation, with significant increases in IGFBP2, IGFBP3, IGFBP4, IGFBP5, and IGFBP7 (p<0.0001) (**Supplementary Figures S1B**–**H**). Additionally, we investigated the correlation between IGFBPs and the clinical traits shown in **Figures 3A**–**G**. For example, IGFBP2, IGFBP3, and IGFBP5 correlated with various malignant subtypes of clinical traits, and all IGFBP expression correlated with pathological grade and treatment response. The above results suggest that the IGFBP family has an important prognostic value for LGG patients.

### Construction and characterization of IGFBP family risk score

**Figure 4:**
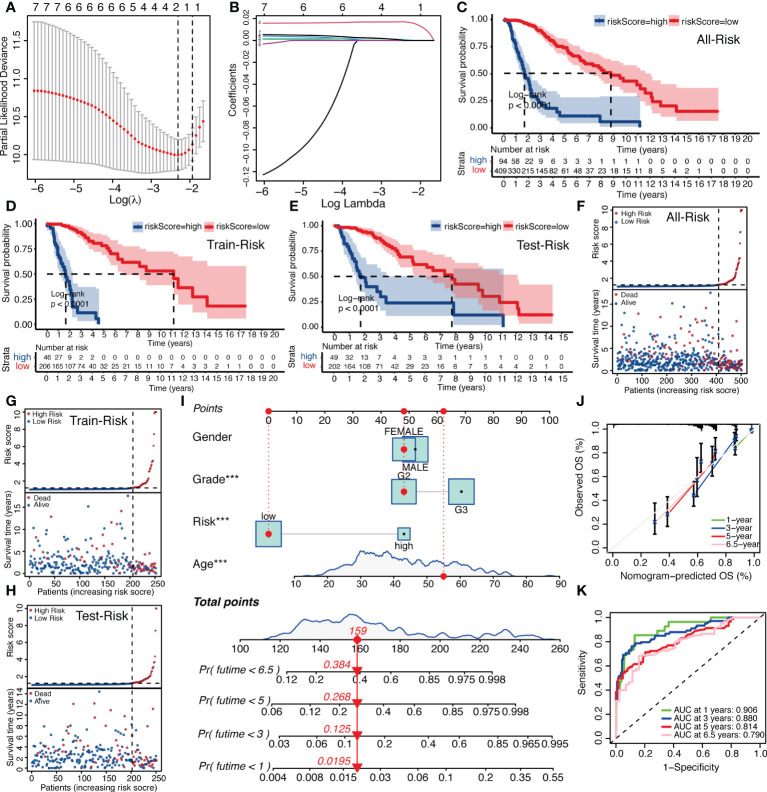
Establishment of IGFBPScore and prognostic prediction. **(A, B)** LASSO regression was performed, calculating the minimum criteria. **(C–E)** Kaplan–Meier curves for the full set, train set and test set in the TCGA glioma cohort. **(F–H)** Risk curve of IGFBPScore for the full set **(F)**, train set **(G)**, and test set **(H)** was plotted using the TCGA glioma cohort. **(I)** Nomogram based on IGFBPScore, age, gender and WHO grade. **(J)** Correlated plot indicated the accuracy of predictive ability. **(K)** ROC curves indicated the risk prediction ability of IGFBPScore at 1, 3, 5, and 6.5 years in the TCGA glioma cohort. For all experiments, ***p<0.001.

Because of the correlation among gene family molecules, we used LASSO COX analysis for compressed estimation to establish the most parsimonious function of the IGFBP family to predict OS of LGG, and the samples were split into a train set (n = 252) and a test set (n = 251). LASSO-COX was used to further determine the principal components of the IGFBP family in the train set, and **Figure 4A** showed the optimal penalty coefficient (log(λ)) of −2.4. On this basis, there were two genes with non-zero coefficients (**Figure 4B**). Therefore, IGFBPScore was constructed and the LASSO coefficients were IGFBP2: 0.0165918577 and IGFBP5: 0.0004779568. The importance of IGFBP2 and IGFBP5 in grouping was also confirmed by random forest, univariate and multivariate Cox regression analysis. In addition, we found that the risk scores of the tumor groups were all significantly higher than those of the normal group, and the risk score of GBM was the highest. Surprisingly, we found that the risk score of the LGG-IDHwt group was significantly higher than that of the LGG-IDHmut group (**Supplementary Figures S2A**–**D**). Next, we split into high-and low-risk groups by the cut-off values of IGFBPScore, with the high-risk subgroup having OS in the total set, train set, and test set (**Figures 4C**–**E**). According to the distribution of IGFBPScore and survival status, suggesting a significantly higher number in deaths of the high-risk subgroup (**Figures 4F**–**H**). The same results were verified using the CGGA database (**Supplementary Figures S2E**, **F**). All of these results suggest that higher IGFBPScores are related to a poor prognosis of LGG. Then we created a nomo-gram using IGFBPScores risk, WHO classification, gender, and age to predict OS in LGG patients (**Figure 4I**), and the calibration plots showed perfect agreement between 1, 3, 5, and 6.5 year observations and the predictions (**Figure 4J**). To assess the predictive ability of this nomagram model for survival, we performed ROC analysis for the LGG cohort, and the AUC values were greater than 0.79 for survival prediction at 1, 3, 5, and 6.5 years, indicating the nomogram had great predictive ability for prognosis (**Figure 4K**).

**Figure 5:**
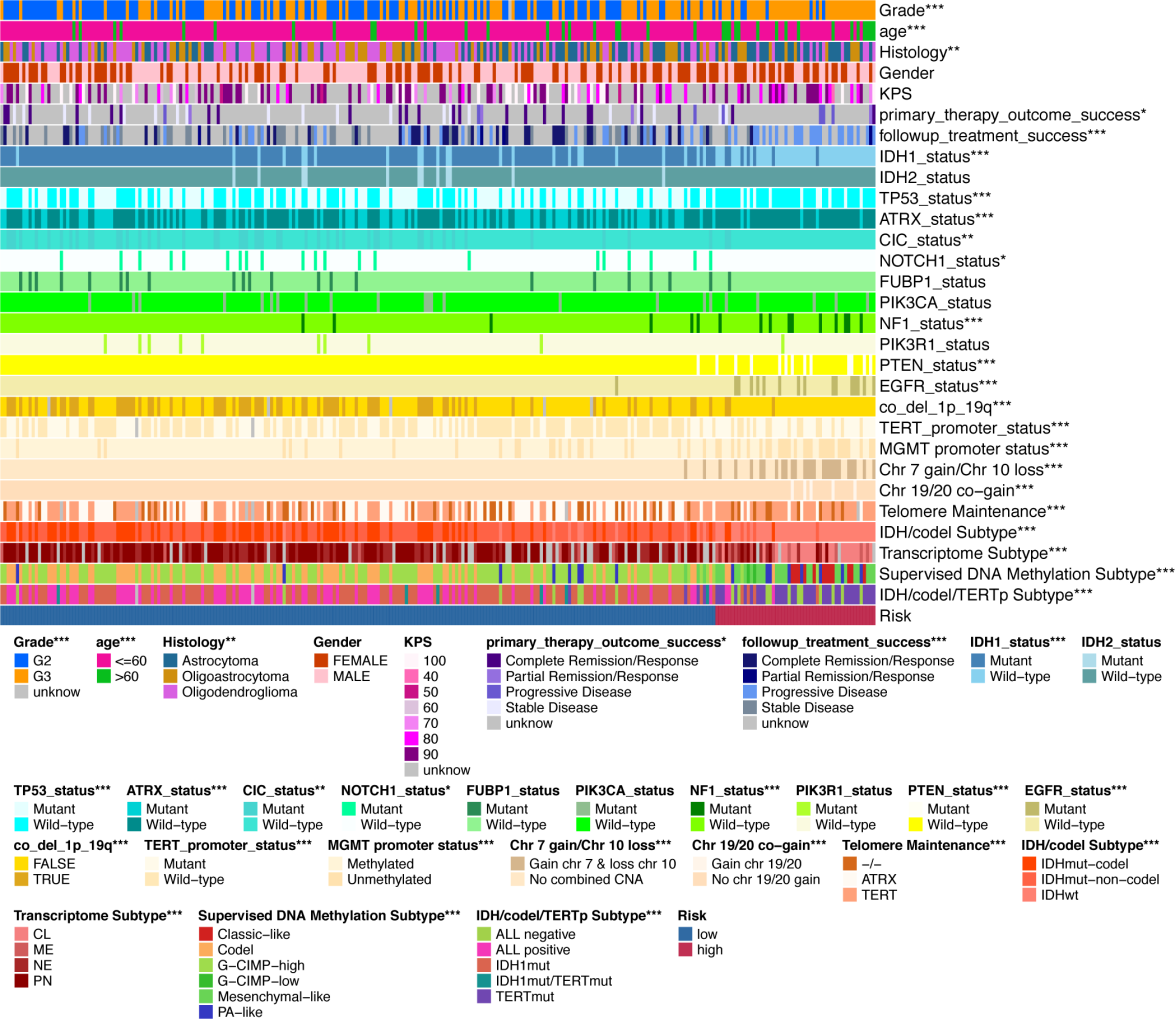
Validation of the clinical value of the IGFBPScore. Correlation heatmap of the IGFBPScore versus common clinical traits, therapeutic response, and molecular subtypes in the TCGA glioma cohort. For all experiments, *p<0.05, **p<0.01, ***p<0.001.

Finally, we used the TCGA LGG cohort to correlate IGFBPScores with multiple tumor traits, including clinical traits, molecular subtypes, and sensitivity to mainstream treatments (**Figure 5**). Clinical traits of advanced age, high-grade, and astrocytes were notably enriched in high-IGFBPScores subgroup; patients with disease progression after the first treatment and at follow-up were notably gathered in high-IGFBPScores subgroup. Molecular subtypes of IDH1 wild type, TP53 wild type, ATRX wild type, CIC wild type, NOTCH1 wild type, NF1 mutant, PTEN mutant, and EGFR mutant were significantly clustered in the high-IGFBPScore subgroup and the high-IGFBPScore subgroup were also significantly enriched in the chromosome 19/20 co-acquired, chromosome 7 acquired 10 deletion, and unaccompanied 1p19q chromosome co-deletion groups, and were also significantly enriched in the TERT-maintained telomeres, TERT promoter mutation, and MGMT promoter non-methylation groups. Most importantly, we also analyzed the four commonly used molecular subtypes and showed the high-IGFBPScore subgroup was also notably enriched in IDH wild-type, classic-like, mesenchymal-like, hypomethylated, and TERT mutation groups. Using the CGGA database, we found that the risk score was also strongly correlated with tumor grade, recurrence, and molecular subtype (**Supplementary Figure S2G**). Taken together, multiple prognostic indicators and tumor characteristics suggested that the high-IGFBPScores subgroup was closely related to the poorer prognosis of LGG.

### Tumorigenesis differences between high-and low-IGFBPScores subgroups

**Figure 6:**
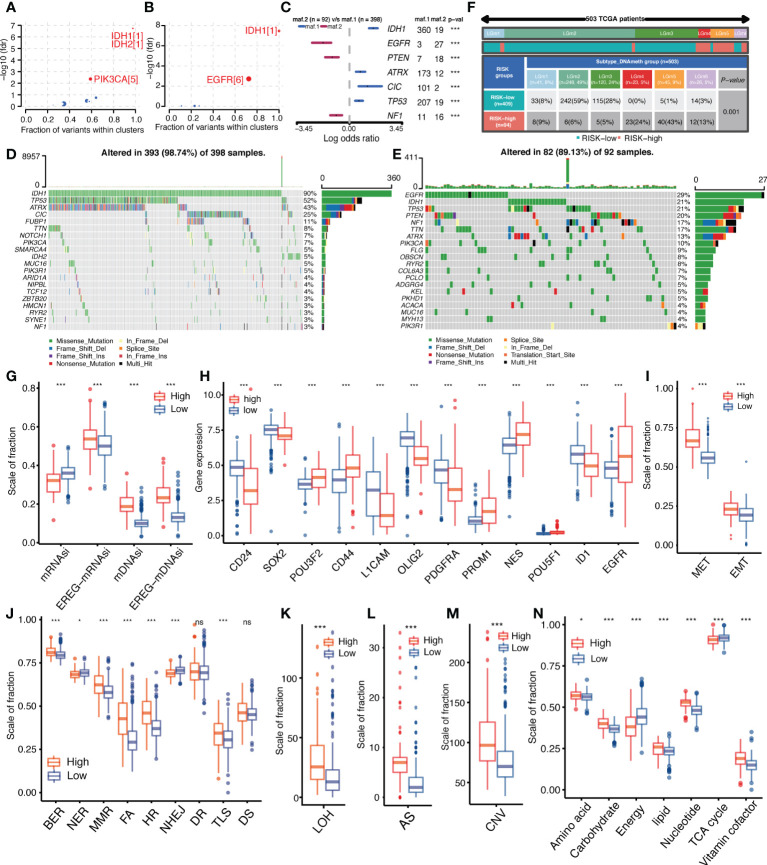
Relationship between IGFBPScore and the development of glioma. **(A, B)** Bubble plot showed the driver genes of High-risk and Low-risk expression groups. **(C)** Forest plot showed the differential mutation patterns in the High-risk and Low-risk groups. **(D, E)** Somatic mutation waterfall plots were created based on High-risk and Low-risk groups. **(F)** Pan-glioma methylation subtypes in High-risk and Low-risk groups. **(G–I)** Differences in stemness index **(G)**, markers of glioma stem cells (**H**), and EMT versus MET phenotypes **(I)** between High-risk and Low-risk groups. **(J–M)** Differences in DNA damage repair pathways (**J**), LOH (**K**), AS (**L**), and CNV (**M**) between High-risk and Low-risk groups. **(N)** Differences in the metabolic levels of seven major substances between the High-risk and Low-risk groups. EMT, epithelial–mesenchymal transition; MET, mesenchymal–epithelial transition, BER, base excision repair; MMR, mismatch repair; FA, Fanconi anemia; HR, homology-dependent recombination; NER, nucleotide excision repair; NHEJ, non-homologous end joining; DR, direct repair; TLS, translesion synthesis; DS, damage sensor; LOH, loss of heterozygosity; CNV, copy number variations; AS, aneuploid score; TAC, tricarboxylic acid cycle. For all experiments, mean rank, *p<0.05, ***p<0.001. ns, no significance.

To explore genomic alterations underlying tumor development and abnormal biological behavior, the “maftools” package was used to analyze differences in driver mutations, mutation patterns, and major somatic mutations between high-and low-IGFBPScores subgroups of LGG. As shown in **Figures 6A**, **B**, both subgroups had IDH mutations as initiating factors, but the differential driver mutation in the low-IGFBPScore subgroup was PIK3CA, while in the high-IGFBPScore subgroup was EGFR, and the mutation patterns between the two subgroups were significantly different, with mutations in EGFR, PTEN, and NF1 predominating in the high-IGFBPScore subgroup, while mutations in IDH1, CIC, TPP53, and ATRX were prominent in the low-IGFBPScores subgroup (**Figure 6C**). Additionally, the overall somatic mutation distribution waterfall also verified the above mutation pattern (**Figures 6D**, **E**), and all mutation genes with significant differences were shown by histogram (**Supplementary Figure S3**). As epigenetics plays a non-negligible role in tumorigenesis as well, we continued to explore the distribution of methylation subtypes in pan-glioma, as shown in **Figure 6F**. The distribution in LGm4 and LGm5 was more in the high-IGFBPScore subgroup, while the distribution in the LGm2 and LGm3 groups was more in the low-IGFBPScore subgroup. It was shown that the LGm1/LGm2/LGm3 group is driven by IDH mutations and exhibits genome-wide hypermethylation, whereas the LGm4/LGm5/LGm6 group is IDH wild-type and is associated with genome-wide hypomethylation (13). In summary, the low-risk group mainly exhibited IDH mutations and hypermethylation, while the high-risk group exhibited IDH wild-type and hypomethylation.

Additionally, the presence and stemness of stem cells are important factors in tumorigenesis. We analyzed the correlation between IGFBPScore and the stemness of glioma cells using four stemness indices: mRNAsi, EREG-mRNAsi, mDNAsi, and EREG-mDNAsi. The results are shown in **Figure 6G**. mDNAsi and EREG-mDNAsi showed significant elevation in high-IGFBPScore subgroup, suggesting high-IGFBPScore was associated with higher stemness of glioma cells, while mRNAsi and mDNAsi showed opposite correlation in glioma. Further analysis of markers of glioma stem cells (GSCs) was performed, including surface markers, spectral markers, and transcription factors (TFs), where among cell surface markers, CD24 was highest in NPC-like cells, CD133 in OPC-like cells, EGFR in AC-like cells, and CD44 in MES-like cells, and among TFs and spectral markers, NES showed a significant bias toward AC-like cells (14). The results are shown in **Figure 6H**. The expression of POU3F2, CD44, PROM1, NES, POU5F1, and EGFR was elevated in the high-IGFBPScore subgroup, while CD24, SOX2, L1CAM, OLIG2, PDGFRA, and ID1 was elevated in the low-elevated subgroup. Based on the markers and GSC types, our findings suggest that AC-like cells, OPC-like cells, and MES-like cells were predominantly present in the high-IGFBPScore subgroup, while NPC-like malignant cells were predominantly present in the low-IGFBPScore subgroup. Additionally, MET and EMT are also important processes for the formation and colonization of stem cells, which we investigated, and the results are shown in **Figure 6I**. Both EMT and MET processes were enhanced in the high-IGFBPScore subgroup, especially MET, indicating that these cells can switch between a quiescent, dormant state and a migratory, mesenchymal-like state. In conclusion, the tumor cells in the high-IGFBPScore subgroup had higher stemness.

### Tumor malignancy phenotype between high and low-IGFBPScore subgroups

Both genetic instability and metabolic disorders are important features of tumors. Among them, the DDR pathway is involved in the process of gene stability as well as gene mutation. To further investigate the relationship between IGFBPScore and genomic stability in LGG, we first analyzed the difference in DDR function between the high-IGFBPScore and low-IGFBPScore subgroups, and the results are shown in **Figure 6J**. Compared with the low-IGFBPScores subgroup, the high-IGFBPScores subgroup showed significantly enhanced BER, MMR, FA, HR, and TLS processes, while the NER and NHEJ processes were significantly weakened. To verify whether altered DDR pathway function between the two subgroups led to the phenotypes of genetic instability, we further evaluated the relationship between IGFBPScore and DNA damage-related phenotypes, as shown in **Figures 6K–M**, where LOH, AS, and CNV were significantly higher in the high-IGFBPScores subgroup than in the low-IGFBPScores subgroup. Taken together, the results of this study suggest that genetic instability was greater in the high-risk group than in the low-risk group. We then proceeded to explore the relationship between IGFBPScore and metabolism in LGG, and the results are shown in **Figure 6N**, including amino acid metabolism, carbohydrate metabolism, energy metabolism, lipid metabolism, nucleotide metabolism, and vitamin coenzyme factor. The 7 metabolic pathways were significantly different between the high- and low-IGFBPScore subgroups, and all were elevated except for energy metabolism and tricarboxylic acid cycle, which were significantly lower in the high-IGFBPScore subgroup.

### TME component and Immunity status differences between high and low-IGFBPScore subgroups

**Figure 7:**
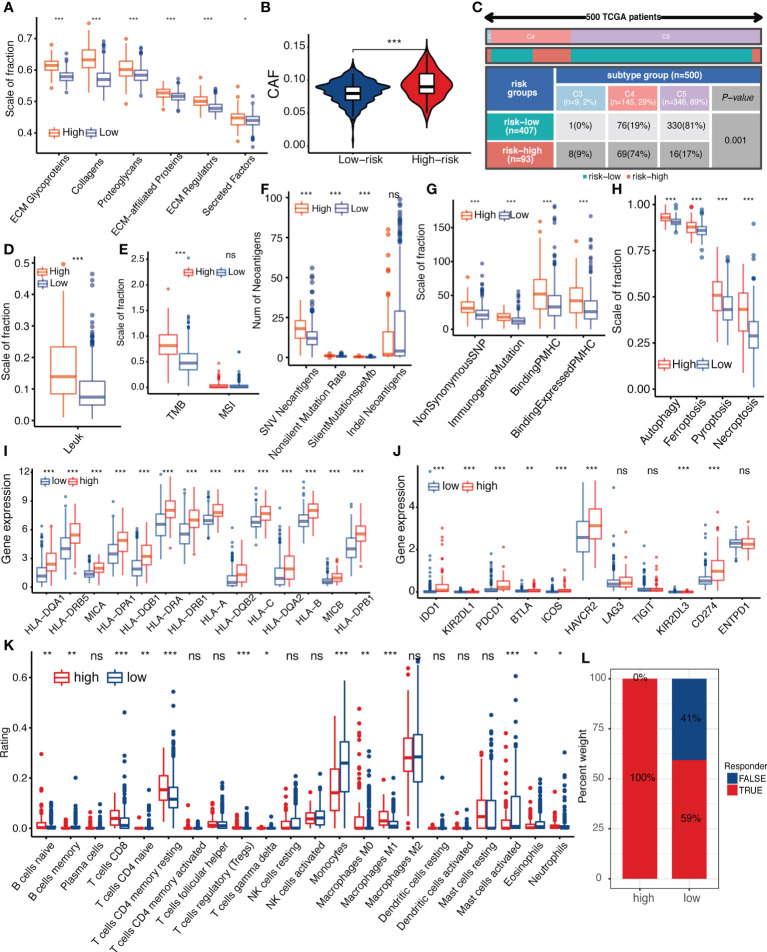
Relationship between IGFBPScore and the tumor microenviroment. **(A)** Differences in extracellular matrix components and **(B)** cancer-associated fibroblasts in High-risk and Low-risk groups. **(C)** Proportion of pan-cancer immune subtypes and **(D)** leukocyte fraction in High-risk and Low-risk groups. (**E–H**) Differences in **(E)** TMB, MSI, **(F)** SNV neoantigen count, **(G)** antigenic peptide-major histocompatibility complex, and **(H)** immunogenic death of High-risk and Low-risk groups. **(I)** Differences in the expression of major histocompatibility complexes and **(J)** immune checkpoints between Low-risk and High-risk groups. **(K)** Differences in tumor-infiltrated immune cells between High-risk and Low-risk groups. (**L**) Differences in response to immunotherapy between High-risk and Low-risk groups. TMB, tumor mutational burden; MSI, microsatellite instability; CAFs, cancer-associated stromal cells; LF, leukocyte fraction; SNV, single nucleotide variants; indel, insertional deletion mutations; MHV, major histocompatibility complex-related genes; pMHC, MHC-binding SNV-derived peptides. For all experiments, mean rank, *p<0.05, **p<0.01, ***p<0.001. ns, no significance.

To investigate the correlation between IGFBPScore and stromal components in the TME of LGG, we first analyzed the correlation between extracellular matrix and IGFBPScore, and the results are shown in **Figure 7A**, where all components of ECM were elevated in the high-IGFBPScore subgroup significantly. Also, cancer-associated stromal cells (CAFs) were similarly elevated in the high-IGFBPScore subgroup (**Figure 7B**). Additionally, among other stromal cells, MSCs, endothelial cells, and lymphatic vessel endothelial cells were also significantly elevated in the high-IGFBPScore subgroup (**Supplementary Figure S1A**). The above results suggest that elevated IGFBPScore was associated with increased stromal cells and increased ECM, exhibiting a high stromal phenotype. Next, we continued to explore the relationship between IGFBPScore and immune components and immune status in LGG TME. First, we analyzed the overall immunophenotype, and the results are shown in **Figure 7C**, with C4 predominating in the high-IGFBPScore subgroup and the C5 phenotype predominating in the low-IGFBPScore subgroup. Studies have demonstrated that the C4 phenotype is lymphocyte-depleted, while C5 is immune-quiet and shows the lowest lymphocytes. The prognosis of the C5 phenotype is better compared to C4 (17). Next, we analyzed the overall leukocyte fraction, immune molecules, and immune pathways. We found that leukocytes, immunostimulatory molecules, and immunosuppressive molecules were significantly higher in the high-IGFBPScore subgroup compared to the low-IGFBPScore subgroup, and immune pathways were more active (**Figure 7D**, **Supplementary Figures S4B**–**D**). Since enhanced immunosuppression is also a compensatory response to enhanced immune activation, tumor anti-inflammatory factors are simultaneously elevated in high inflammatory responses (18). In summary, we concluded that the immune component was higher and the immune status was more active in the high-IGFBPScore subgroup and was associated with poor prognosis.

Next, we proceeded to analyze immune antigenicity and antigen presentation, as shown in **Figures 7E**–**I**. Tumor mutation burden (TMB), single nucleotide variant (SNV) neoantigens, including those generated by insertional deletion mutations (indel), and silent and non-silent mutation rates, all major histocompatibility complex (MHC)-associated genes, and MHC-bound SNV-derived peptide (pMHC) were significantly higher in the high-IGFBPScore subgroup, and immunogenic death such as autophagy, ferroptosis, pyroptosis, and necroptosis were also significantly higher in the high-IGFBPScore subgroup, and the above results suggested that there was strong antigenicity as well as antigenic presentation in high-IGFBPScore subgroup. Additionally, by comparing the immune infiltration and immune checkpoints between the high and low-IGFBPScore subgroups, we found most immune cells were significantly different between the two subgroups, where most immune cells were increased in the high-IGFBPScore subgroup, e.g., increased CD4 memory T and CD8 T, while the low-IGFBPScore subgroup mainly showed significantly increased monocytes as well as MDSC (**Figure 7K**, **Supplementary Figure S4E**). Nevertheless, our results showed that the expression of immune checkpoints TIM-3 and PD-1 was also higher in the high-IGFBPScore subgroup (**Figure 7J**), immune damage was enhanced (**Figure S4F**), and so was the response to immunostimulatory therapy (**Figure 7L**), and there were little variations in immune escape between the two subgroups (**Supplementary Figure S4G**). In summary, we concluded that the overall immune response was stronger in the high-IGFBPScore subgroup relative to the low-IGFBPScore subgroup.

## Discussion

Since the IGFBP family plays an important role in tumors, we first validated the clinical significance of the IGFBP family in LGG, and then we simplified and downscaled by lasso cox analysis to obtain the IGFBPScore as a principal component of the IGFBP family. We validated our results by showing that the IGFBPScore is important for prognosis prediction, subtype assessment, and treatment sensitivity determination in LGG patients. Additionally, we analyzed the role of IGFBPScore, representing the IGFBP family, in the development of LGG as well as in the composition of the tumor microenvironment. Our results suggested that tumors with high or low IGFBPScore had different mutational patterns, different epigenetic features, and differences in starting stem cell types in ontogenesis. Furthermore, high IGFBPScore was associated with high stemness, high inflammation, high ECM phenotype and metabolic disorders, leading to poor prognosis.

The IGFBP family is one of the most important families of ECM regulatory proteins, and the intranuclear roles of IGFBPs in transcriptional regulation, induction of apoptosis, and DNA damage repair suggest their close involvement in tumor development, progression, and resistance to therapy (8). It was shown that IGFBP3, IGFBP5, and IGFBP7 were highly expressed in glioma and were associated with higher tumor grade and poorer survival, consistent with our study (9, 10, 19). Additionally, Cai et al. showed that IGFBP2 expression was upregulated in high-grade gliomas and downregulated in IDH mutant gliomas (20), supporting our results. Moreover, when cancer cells are driven by ligands, the cells are driven by ligand-dependent IGF1R activation, which can lead to increased subsequent local IGFBP secretion (8). Additionally, IGFBP2 can promote the formation of angiogenic mimics by regulating the expression of CD144 and MMP2 in glioma as well as enhancing nuclear EGFR-STAT3 signaling, which can be used as a therapeutic target for glioma (11, 21). Our findings collectively suggested that most IGFBP family members were dysregulated and had important biological functions in LGG, which were associated with poor prognosis and had important research value.

In this study, we first constructed the risk score model of the IGFBP family in LGG, and in addition to its important clinical application, we explored the biological mechanisms of LGG. First, we analyzed the gene mutations that underlie cancer development, and our results showed that the high IGFBPScore group had mutation patterns of EGFR, PTEN, and NF1, while the main mutation patterns of the low IGFBPScore group were IDH1, ATRX, CIC, and TP53, and previous studies showed that tumors with IDH mutations were usually accompanied by TP53, CIC, and ATRX mutations, a marker of good prognosis, while PTEN, EGFR, and NF1 mutations were characteristic of IDH wild-type gliomas, which were similar to glioblastoma (GBM) in terms of molecular features and clinical behavior (22–25), in agreement with our results. Also, IDH mutations were accompanied by increased methylation levels, and in gliomas the G-CIMP-low group was associated with a poorer prognosis (26), also supporting our results. In addition, GSCs are an important factor contributing to gliomagenesis, and our findings suggest that mDNAsi was higher in people with a high IGFBPScore. Previous studies have shown that mDNAsi was positively correlated with advanced pathological grade of gliomas, driven mainly by hypomethylation associated with stemness, and was highest in highly aggressive gliomas characterized by mesenchymal-like subtypes (15). Second, based on this, we further investigated the types of GSCs in different risk groups, and our results suggested that AC-like cells, OPC-like cells, and MES-like cells were mainly present in the high-risk group, while only NPC-like malignant cells were more frequent in the low-risk group. Among them, MES-like, NPC-like, and OPC-like cells were associated with recurrence and increased grade and aggressiveness of glioma, with stem cell diversity in the high-risk group and only NPC-like malignant cells in the low-risk group (14), implying that it was GSC-restricted and may limit the growth rate of tumors. In conclusion, we suggest that the high-IGFBPScore group developed from gliomas with advanced glioma mutation patterns and high stemness and stem cell diversity.

Studies have shown that the composition of TME in brain tumors is very unique and is influenced by molecular features (27). We found that in the pan-cancer immunophenotype, the low-risk group was mainly concentrated in the C5 subtype. It has been shown that 80% of IDH mutations were enriched in C5 (16), and in fact, IDH mutations can decrease tumor-associated immune cells and improve prognosis by reducing leukocyte chemotaxis (28, 29), while the high-risk group was mainly concentrated in the C4 phenotype and contained a higher level of immune antigenicity, immunomodulation, and leukocyte infiltration, which was associated with a poorer prognosis. In addition to the composition of immune cells, the structure of the brain extracellular matrix (ECM) seems to help distinguish the characteristics of LGG. Compared to other organs and tissues, the structure and composition of the brain ECM is unique, containing mainly heparan sulfate proteoglycans (HSPG) and hyaluronic acid (HA) (30). Studies have shown that HSPG production is significantly increased in gliomas and that dense ECM leads to hypoxia and tumor aggressiveness (31). A recent study showed that ECM stiffness was associated with an increase in glioma grade, while ECM stiffness and aggressiveness showed an opposite relationship to IDH mutation (32). Consistent with our study, all matrix proteins were significantly elevated in the high-risk group, and cancer-associated fibroblasts were also significantly increased. Taken together, we concluded that high IGFBPScore risk had a high inflammatory and high extracellular matrix phenotype that contributes to multiple biological processes and glioma progression.

Previous studies have shown that in the human CNS, accumulation of IGFBP2 is observed in activated macrophages (33). These IGFBP2-positive macrophages accumulate near the center of necrosis (34). Additionally, high IGFBP2 can act as a regulator of PD-L1 *via* the EGFR–STAT3 pathway and is associated with shorter overall survival in melanomas (35), while it has also been shown that IGFBP2 can activate the EGFR-STAT3 pathway in glioma (21). In conclusion, there is growing evidence that IGFBP2 is associated with cancer-related immune responses. Additionally, iGFBP2 has been overexpressed within the stem cell compartment of GBM as an important factor in the clonal expansion and proliferative properties of glioma stem cells (36). Furthermore, previous studies have shown that low expression levels of IGFBP2 are associated with a general hyper-methylation phenotype and improved survival in gliomas (37). Moreover, as an ECM regulatory protein, the extracellular matrix densification caused by increased IGFBPs is an important factor in tumor progression (38). IGFBP2 is considered as one of the three most promising blood markers for glioma (39), and future studies will focus more on the mechanisms of IGFBP in glioma.

This study still has some limitations. First, our study mainly demonstrated correlations, which we will validate with basic experiments in a follow-up study. Secondly, our study was performed on the basis of the RNA-seq matrix of whole cancer tissues, thus the intra-tumoral heterogeneity of LGG could not be assessed. Finally, we used a functional classification score (FCS) approach to quantify phenotypes and pathways, but FCS analyzes each pathway independently, which may lead to significant enrichment of individual pathways due to gene overlap because the same gene may be involved in multiple pathways.

In this article, we established for the first time an IGFBPScore for LGG using the clinically important IGFBP family, which contributes to prognostic prediction, subtype assessment, and determination of therapeutic sensitivity in LGG patients.

## Conclusion

Furthermore, our results suggested that high IGFBPScore was mainly associated with tumors with GBM mutation patterns, hypomethylation levels, and stem cell pleiotropy, and with a malignant phenotype exhibiting high genetic instability, metabolic disorders, high inflammation, and high ECM. Our study provides a comprehensive summary analysis of the clinical significance and biological mechanisms of the IGFBP family in LGG, providing an important theoretical basis for targeted therapy of glioma.

## Data availability statement

The original contributions presented in the study are included in the article/**Supplementary Material**. Further inquiries can be directed to the corresponding authors.

## Author contributions

ZL conceived and designed the study. JZ, WF, and NW were responsible for data collection, analysis, and checking. HZ, JD, and XY collated the data. ZL wrote the original draft of the manuscript, which was reviewed and revised by HJ and SM. SH and JW supervised the study. All authors listed have made a substantial, direct, and intellectual contribution to the work and approved it for publication.

## Funding

This work was funded by the National Natural Science Foundation of China (No. 61575058).

## Acknowledgments

The authors gratefully acknowledge databases including TCGA, CGGA, GTEx, UCSC, GlioVis, and many others for offering convenient access to datasets and user-friendly online analysis.

## Conflict of interest

The authors declare that the research was conducted in the absence of any commercial or financial relationships that could be construed as a potential conflict of interest.

## Publisher’s note

All claims expressed in this article are solely those of the authors and do not necessarily represent those of their affiliated organizations, or those of the publisher, the editors and the reviewers. Any product that may be evaluated in this article, or claim that may be made by its manufacturer, is not guaranteed or endorsed by the publisher.

## Supplementary material

Supplementary Figure 1 Clinical significance and validation of riskscores. **(A)** Heatmap of differential expression analysis of IGFBP family between TCGA IDHmut glioma and IDHwt glioma cohort. **(B-H)** Kaplan-Meier survival analysis of IGFBP family was performed using CGGA glioma cohort. *p<0.05, **p<0.01, ***p<0.001.

Supplementary Figure 2 Validation of composition and clinical significance of riskscores. **(A)** Random forest is used to analyze the weight of grouping variables using TCGA cohort. **(B)** Riskscores variance analysis for normal cerebral cortex, low-grade glioma IDHmut group, low-grade glioma IDHwt group and glioblastoma using TCGA cohort. **(C-D)** Hazard Ratio of IGFBP family was determined by univariate and multivariate cox analysis using TCGA cohort. **(E)** Kaplan-Meier curves for validation using the CGGA cohort. (F) Risk curve of IGFBPScore was plotted using the CGGA cohort. **(G)** Correlation heatmap of the IGFBPScore in the CGGA cohort. For all experiments, *p<0.05, **p<0.01, ***p<0.001.

Supplementary Figure 3 Comparison of mutated genes in high- and low- riskscore groups. **(A-P)** The histogram showed mutanted genes with significant differences of the waterfall plots.

Supplementary Figure 4 Relationship between IGFBPScore and other immune characteristics. **(A)** Differences in stromal cells between Low-risk and High-risk groups. **(B-D)** Differences in immune-stimulatory genes **(B)** and immune-suppressive genes **(C)** tumor microenvironment-related pathways **(D)** of Low-risk and High-risk groups. (E) Differences in MDSC between Low-risk and High-risk groups. Differences in **(F)** immune dysfunction and **(G)** immune escape between Low-risk and High-risk groups. Myeloid derived suppressor cell (MDSC), For all experiments, mean rank, *p<0.05, **p<0.01, ***p<0.001.
