# Supplementary figures and images for: IGFBPs were associated with stemness, inflammation, extracellular matrix remodeling and poor prognosis of low-grade glioma
Source: Front Endocrinol (Lausanne). 2022 Aug 3;13:943300. doi: 10.3389/fendo.2022.943300 (PMC9381844; doi:10.3389/fendo.2022.943300)

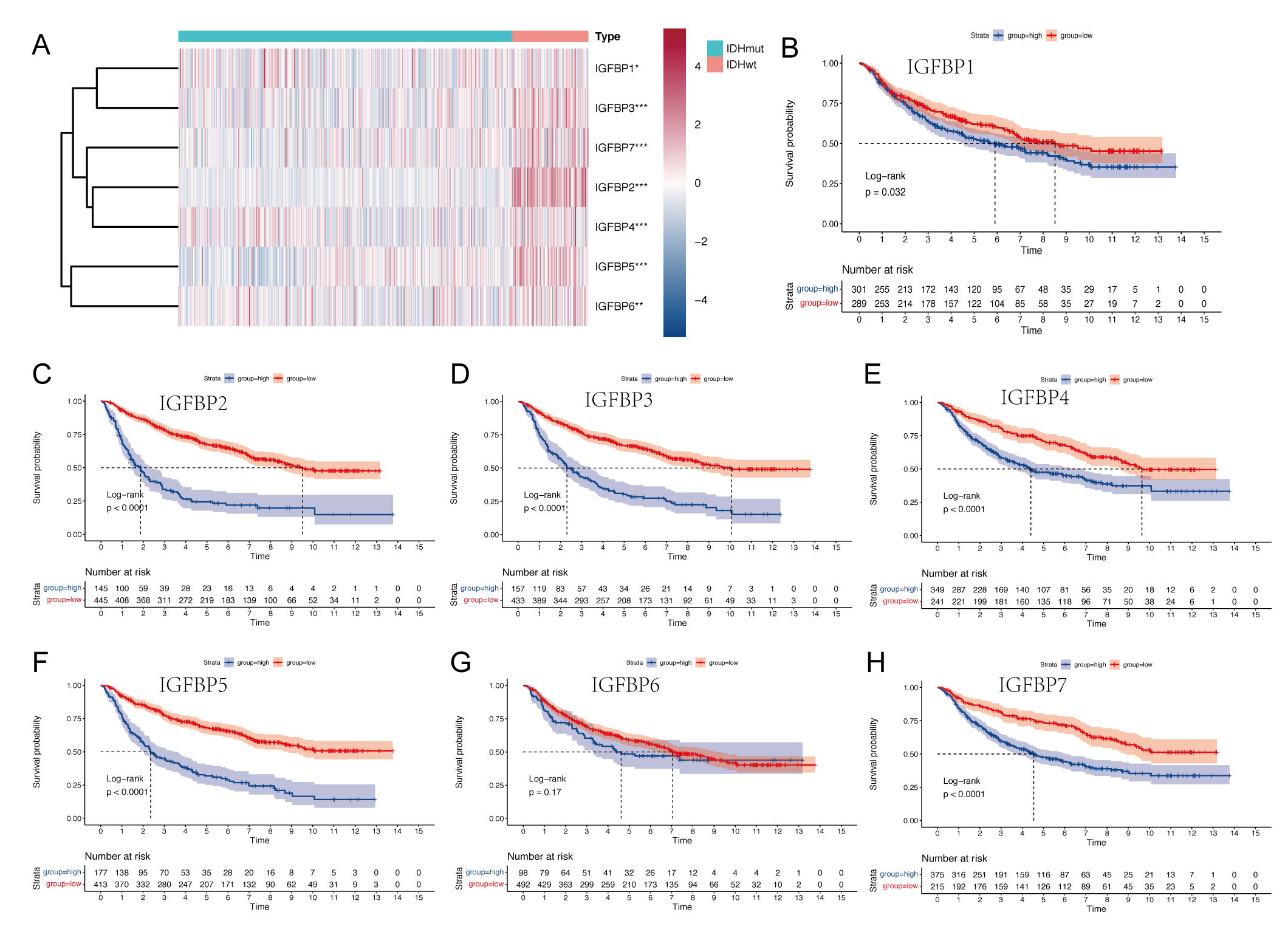

Supplement: Supplementary Figure 1 — Clinical significance and validation of riskscores. (A) Heatmap of differential expression analysis of IGFBP family between TCGA IDHmut glioma and IDHwt glioma cohort. (B-H) Kaplan-Meier survival analysis of IGFBP family was performed using CGGA glioma cohort. *p<0.05, **p<0.01, ***p<0.001. [file Image_1.tif]

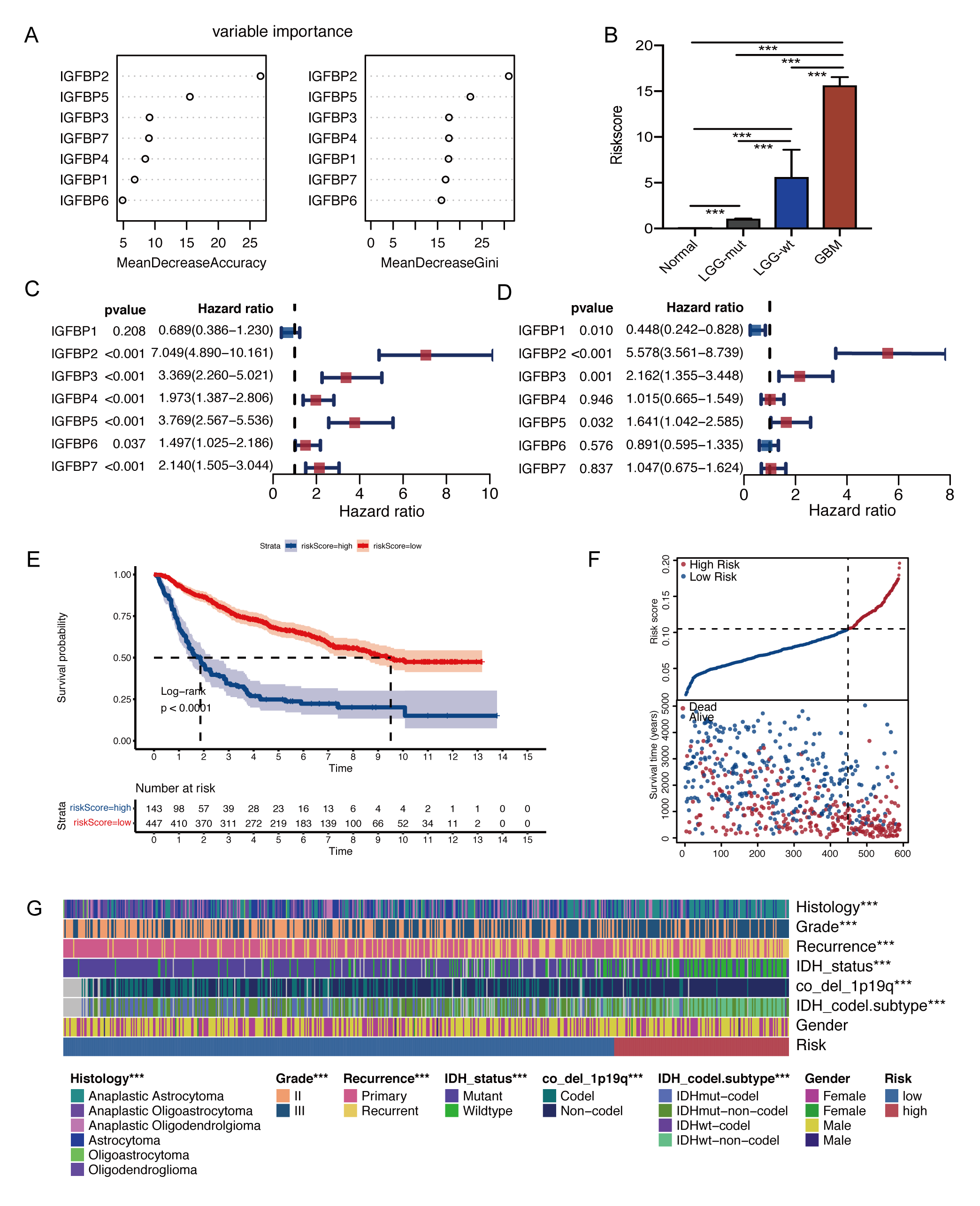

Supplement: Supplementary Figure 2 — Validation of composition and clinical significance of riskscores. (A) Random forest is used to analyze the weight of grouping variables using TCGA cohort. (B) Riskscores variance analysis for normal cerebral cortex, low-grade glioma IDHmut group, low-grade glioma IDHwt group and glioblastoma using TCGA cohort. (C-D) Hazard Ratio of IGFBP family was determined by univariate and multivariate cox analysis using TCGA cohort. (E) Kaplan-Meier curves for validation using the CGGA cohort. (F) Risk curve of IGFBPScore was plotted using the CGGA cohort. (G) Correlation heatmap of the IGFBPScore in the CGGA cohort. For all experiments, *p<0.05, **p<0.01, ***p<0.001. [file Image_2.tif]

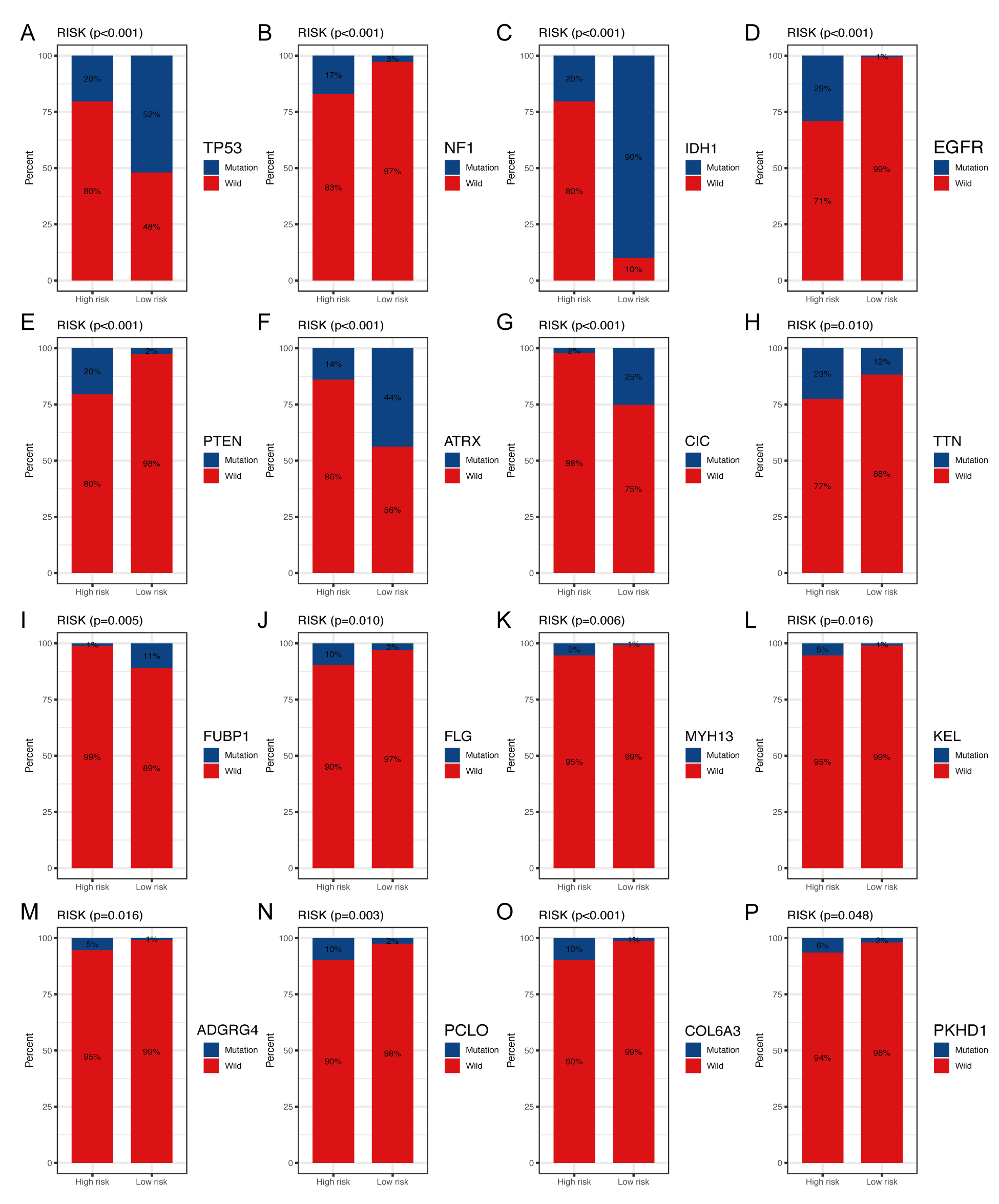

Supplement: Supplementary Figure 3 — Comparison of mutated genes in high- and low- riskscore groups. (A-P) The histogram showed mutanted genes with significant differences of the waterfall plots. [file Image_3.tif]

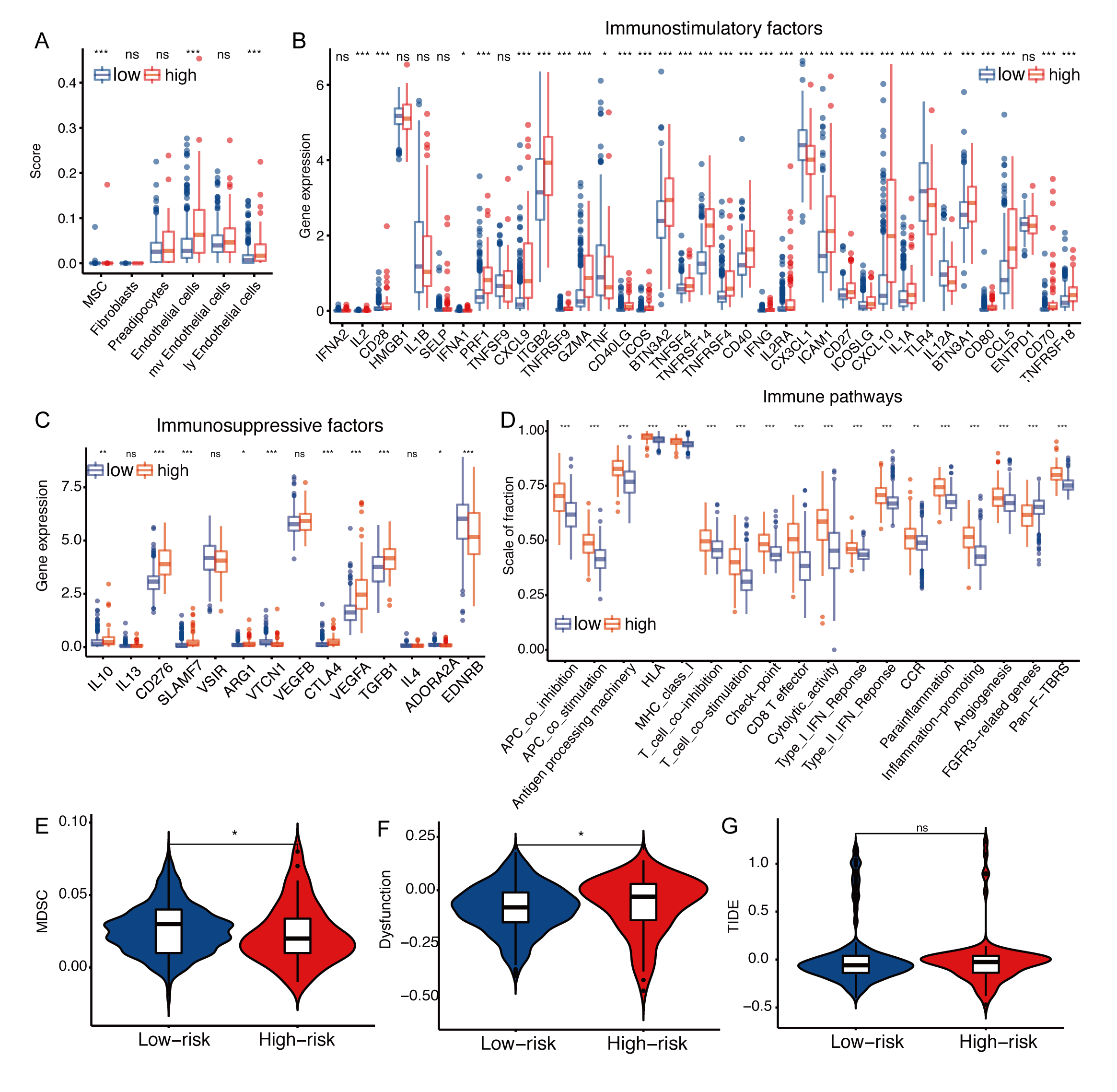

Supplement: Supplementary Figure 4 — Relationship between IGFBPScore and other immune characteristics. (A) Differences in stromal cells between Low-risk and High-risk groups. (B-D) Differences in immune-stimulatory genes (B) and immune-suppressive genes (C) tumor microenvironment-related pathways (D) of Low-risk and High-risk groups. (E) Differences in MDSC between Low-risk and High-risk groups. Differences in (F) immune dysfunction and (G) immune escape between Low-risk and High-risk groups. Myeloid derived suppressor cell (MDSC), For all experiments, mean rank, *p<0.05, **p<0.01, ***p<0.001. [file Image_4.tif]
